# Cold Exposure Exacerbates Allergic Airway Inflammation via Ferroptosis: Evidence from a Murine Model

**DOI:** 10.3390/antiox15010032

**Published:** 2025-12-24

**Authors:** Xiaoping Guo, Chao Wang, Xin Yu, Shanshan Zhang, Haoyu Zheng, Tianqi Liu, Zhili Chen, Guoqiang Wang, Fang Wang

**Affiliations:** 1 Department of Pathogen Biology, College of Basic Medical Sciences, Jilin University, Changchun 130021, China; guoxp23@mails.jlu.edu.cn (X.G.); chao_wang98@sina.cn (C.W.); xyu23@mails.jlu.edu.cn (X.Y.); shanshan23@mails.jlu.edu.cn (S.Z.); zhenghy22@mails.jlu.edu.cn (H.Z.); liutq9922@mails.jlu.edu.cn (T.L.); chenzl9922@mails.jlu.edu.cn (Z.C.); wanggq20@jlu.edu.cn (G.W.); 2 The Medical Basic Research Innovation Center of Airway Disease in North China, Changchun 130021, China; 3 Cross-Disciplinary Innovation Center, Jilin University, Changchun 130021, China; 4 Gene Function and Human Disease Model Organism Sharing Service Platform, Changchun 130021, China; 5 Jilin Provincial International Cooperation Key Laboratory of Pathogen Biology, Changchun 130021, China; 6 Jilin Provincial Key Laboratory of Precision Infectious Diseases, Changchun 130021, China

**Keywords:** allergic airway inflammation, cold exposure, ferroptosis, Ferrostatin-1

## Abstract

Recently, extreme weather has been regarded as a risk factor for exacerbating allergic airway inflammation (AAI), but its underlying mechanism remains unclear. Ferroptosis, an iron-dependent form of regulated cell death driven by lipid peroxidation, has been implicated in various lung diseases. This study investigated whether cold exposure aggravated OVA-induced AAI by promoting ferroptosis. A murine AA model was established using OVA sensitization and challenge. Mice were exposed to cold temperatures (10 °C or 4 °C) for 4 h daily. Ferroptosis was assessed by measuring ferroptosis-related markers (GPX4, ACSL4, FTL), iron deposition (Alcian Blue-Periodic Acid-Schiff Staining), lipid peroxidation (MDA), antioxidant levels (GSH), and mitochondrial ultrastructure (TEM). The ferroptosis inhibitor ferrostatin-1 (Fer-1) was administered to evaluate its protective effects. Airway inflammation, lung function, and histopathology were also analyzed. Cold exposure significantly worsened AA symptoms, including increased Th2 cytokine levels (IL-4, IL-5, IL-13, IL-33), impaired lung function, and enhanced airway remodeling and mucus production. These effects were more pronounced at 4 °C. Cold exposure also induced ferroptosis, as evidenced by decreased GPX4 and FTL, increased ACSL4, elevated iron and MDA levels, reduced GSH, and mitochondrial damage. Treatment with Fer-1 mitigated these changes, alleviating inflammation, improving lung function, and reducing histological damage. Cold exposure exacerbated AAI by inducing ferroptosis in lung tissues. Inhibition of ferroptosis with Fer-1 attenuated these aggravation effects, suggesting ferroptosis as a potential mechanistic link between cold exposure and AAI severity. Targeting ferroptosis might offer a novel therapeutic strategy for mitigating AAI under cold conditions.

## 1. Introduction

Allergic airway inflammation (AAI) is a common respiratory disorder characterized by persistent inflammation of the airways, currently affecting an estimated 358 million individuals globally and representing a substantial worldwide health challenge [1]. From a pathological perspective, its primary features encompass not only this persistent airway inflammation but also structural remodeling of airway tissues, impairment of epithelial barrier integrity, abnormal proliferation of goblet cells, and dysregulated production and secretion of mucus [2]. The pathogenesis of allergic airway inflammation (AAI) is driven by a complex network of inflammatory and immune factors, where a precisely regulated balance among inflammation, immunity, and metabolism is essential for preserving systemic homeostasis [3]. Notably, inflammatory cytokines including IL-4, IL-5, IL-13, and IL-33 are frequently overexpressed in AAI, contributing to the amplification of allergic responses, exacerbation of airway inflammation, and induction of airway hyperresponsiveness [4]. Concomitantly, aberrant lung function parameters such as elevated airway resistance and reduced dynamic compliance are tightly linked to the airway pathological remodeling triggered by these cytokines [5]. It is often triggered by environmental factors such as allergens, pollutants, and climatic conditions [6,7]. Among them, cold exposure is recognized as one of the risk factors for AAI attacks. Epidemiological studies have shown that cold exposure can significantly increase the emergency visit rate and hospitalization rate of AAI patients, but the underlying mechanism remains unclear [8,9].

Ferroptosis is a newly identified type of iron-dependent, regulated cell death [10]. Its molecular mechanisms mainly involve multiple aspects such as iron metabolism imbalance, enhanced lipid peroxidation, and impaired function of the antioxidant system (e.g., glutathione peroxidase 4, GPX4) [11]. An increasing number of studies have demonstrated that ferroptosis plays a key role in the pathophysiological processes of various lung diseases [12]. For instance, in acute lung injury, ferroptosis of alveolar epithelial cells could exacerbate inflammatory responses and lung tissue damage [13]. In chronic obstructive pulmonary disease (COPD), cigarette smoke extract could promote disease progression by inducing ferroptosis in airway epithelial cells [14]. Additionally, recent studies have revealed abnormal iron metabolism and elevated levels of lipid peroxidation products in the airway tissues of AAI patients, suggesting that ferroptosis may be involved in the pathogenesis of AAI [15]. To specifically inhibit this pathway, pharmacological tools such as ferrostatin-1 (Fer-1) have been developed. Fer-1 is a specific ferroptosis inhibitor. It blocks the lipid peroxidation chain reaction by eliminating lipid free radicals, inhibits lipoxygenase, and indirectly regulates iron metabolism to reduce ROS generation, protecting the integrity of cell membranes to prevent ferroptosis [16]. With its high specificity and low toxicity, it is widely used in research on the mechanism of diseases related to ferroptosis. Therefore, leveraging such tools, investigating whether cold exposure exacerbates AAI symptoms by affecting ferroptosis would be of great significance for clarifying the potential mechanisms underlying the impact of climatic factors on AAI and developing new preventive strategies.

In this study, an ovalbumin (OVA)-sensitized and challenged murine model was employed, which recapitulated several hallmark features of AAI, including Th2 inflammation, airway hyperresponsiveness, mucus hypersecretion, and tissue remodeling. Using this model, we established a cold-exposed AAI group and confirmed that cold exposure significantly aggravated AAI symptoms in mice. To explore whether this exacerbation was mediated through ferroptosis, key ferroptosis-related indicators were assessed, including glutathione peroxidase 4 (GPX4) expression and mitochondrial ultrastructure. Furthermore, the ferroptosis inhibitor ferrostatin-1 (Fer-1) was administered to examine whether inhibiting ferroptosis could mitigate the cold-induced worsening of AAI. Collectively, our findings demonstrated that cold exposure exacerbated AAI, at least in part, by enhancing ferroptosis. This study elucidated a mechanistic link among cold exposure, ferroptosis, and AAI progression, offering a novel theoretical foundation and potential therapeutic targets for managing cold exposure-aggravated allergic airway conditions. It is important to note that this model induces a robust, pan-lung allergic-type inflammation, in contrast to the more bronchial-centric pathology often observed in human asthma. Nonetheless, the shared core inflammatory pathways and environmental triggers make this system highly valuable for dissecting mechanisms that may also operate in the more anatomically restricted human disease.

## 2. Materials and Methods

### 2.1. Animal Model Establishment

This study conducted animal experiments using female BALB/c mice (6–8 weeks old). All animal experiments were carried out in accordance with the guidelines approved by the Animal Protection and Use Review Committee of Jilin University and were approved by the Animal Ethics Committee of the School of Basic Medicine, Jilin University (No. 2024-369). All the animals were housed in a Specific Pathogen Free (SPF) environment with controlled temperature (22 ± 2 °C) and humidity (50 ± 10%), and a 12-h light/dark cycle. The grouping of animal experiments was as follows: RT-NC group: room temperature control group; 10-NC group: only the 10 °C exposure treatment group; 4-NC group: only the 4 °C exposure treatment group; RT-AAI group: the group that was sensitized by OVA and challenged at room temperature; 10-AAI group: the group that received 10 °C cold exposure and was sensitized by OVA and challenged; 4-AAI group: the group that received 4 °C cold exposure and was sensitized by OVA and challenged; 4-AAI-Fer-1 group: the group that received 4 °C cold exposure and was sensitized by OVA, followed by intraperitoneal injection of Fer-1. The specific procedures for establishing animal models were as follows: on days 0, 7, and 14, the AAI mice (RT-AAI, 10-AAI, 4-AAI, 4-AAI-Fer-1, *n* = 6) were intraperitoneally injected with Al(OH)_3_ (2.5 μL/g, Cat#60749ES10, Yeasen, Shanghai, China) and OVA (2.5 μg/g, Cat#A5503, Sigma-Aldrich, St. Louis, MO, USA) for sensitization (the NC group was intraperitoneally injected with an equal volume of PBS), and on days 21–23, the AAI mice were challenged with OVA nebulization (the NC group was nebulized with an equal volume of PBS). Mice in the 4-AAI-Fer-1 group received intraperitoneal injections of Fer-1 (1 mg/kg, Cat# HY-100579, MCE, Dallas, TX, USA) on days 14 and 21, respectively. Starting from day 0, the corresponding groups of mice were subjected to low-temperature treatment at 10 °C (10-NC and 10-AAI) or 4 °C (4-NC, 4-AAI, and 4-AAI-Fer-1) for 4 h every day. On day 24, all mice were euthanized and samples were collected.

Animals were euthanized by exsanguination under general anaesthesia with isoflurane (Cat#C153359, Aladdin, Shanghai, China).

### 2.2. Cytokine Detection

The expression levels of IL-4, IL-5, IL-13, and IL-33 in the serum and lung tissues of mice were quantitatively detected using ELISA kits (Cat#EK204, Cat#EK205, Cat#EK213, Cat#EK233, Multi Sciences, Hangzhou, China). All procedures were carried out according to the instructions provided by the manufacturer.

### 2.3. PCR Array and Quantitative Real-Time PCR (qRT-PCR) Validation

Lung tissue total RNA was isolated with TRIzol reagent (Cat#DP424, Tiangen Biotech, Beijing, China), adhering to the manufacturer-provided protocol. Subsequently, cDNA synthesis was executed using a reverse transcription kit, following the recommended procedure. Gene expression profiling was analyzed by Death screening PCR Array (Cat#WC-MRNA0358-M, Wcgene Biotech, Shanghai, China), according to the manufacturer’s protocol. For gene expression quantification, quantitative PCR was conducted with FastStart Universal SYBR Green Master premix (Cat#04913914001, Sigma-Aldrich, USA) and a Fast qPCR System. The standard curve method was applied for data analysis. The primer sequences utilized in this study are detailed in Table S1.

### 2.4. Lung Function Measurement

Lung function was assessed with the Buxco lung function testing system, as detailed in prior research [2]. Briefly, mice were anesthetized and underwent tracheal intubation, following which the endotracheal tube was attached to the system’s spirometer, barometric, and pressure transducers. Airway responsiveness was subsequently evaluated after exposure to escalating concentrations (0, 3.125, 6.25, 12.5, 25 mg/mL) of Acetyl-β-chloromethylcholine (Cat#A2251, Sigma-Aldrich, USA), and lung parameters were automatically recorded by the spirometry system.

### 2.5. Malondialdehyde (MDA) and Glutathione (GSH) Assay

To evaluate the degree of lipid peroxidation, the MDA and GSH levels in the lung tissue of mice were quantitatively determined using the lipid peroxidation MDA (Cat#A003-1-2, Nanjing Jiancheng, Bioengineering Institute, Nanjing, China) and GSH (Cat#S0053, Beyotime, Shanghai, China) assay kits. The experiment was carried out strictly in accordance with the manufacturer’s instructions.

### 2.6. Colorimetric Assay (Ferrozine Method) for Iron Ion Content Detection

To quantify ferrous iron (Fe^2+^) levels in biological samples, a colorimetric assay based on the formation of a purple-colored complex between ferrous ions and Ferene-S was employed (Cat#BC5415, Solarbio, Beijing, China). The resulting complex exhibits a characteristic absorption peak at 562 nm, and its absorbance was directly proportional to the Fe^2+^ concentration. The absorbance was measured spectrophotometrically, allowing for the determination of ferrous iron level tissue homogenates.

### 2.7. Western Blot

Mouse lung tissues were harvested into RIPA lysis buffer (Cat#R0010, Solarbio, China) and homogenized thoroughly. Proteins were separated by SDS-PAGE, transferred to a PVDF membrane (Cat#IPVH00005, Merck Millipore, Darmstadt, Germany), and blocked with 5% BSA (Cat#ST023, Beyotime, China) for 2 h. The membrane was then incubated with primary antibodies at 4 °C overnight, washed with TBST, and incubated with HRP-labeled secondary antibody (Cat#RS0008, Immunoway, San Jose, CA, USA) for 1 h. The primary antibodies used were: Anti-GPX4 pAb (Cat#30388-1-AP, Proteintech, China), Anti-ACSL4 pAb (Cat#22401-1-AP, Proteintech, Wuhan, China), Anti-FTL pAb (Cat#10727-1-AP, Proteintech, China), and Anti-β-actin pAb (Cat# 20536-1-AP, Proteintech, China). Protein signals were detected and quantified using ImageJ (version 1.53t).

### 2.8. Transmission Electron Microscope (TEM)

Lung tissues fixed in 2.5% glutaraldehyde were rinsed, post-fixed in 1% osmium tetroxide, dehydrated (gradient ethanol), infiltrated, and embedded in epoxy resin. Ultrathin sections were stained with uranyl acetate and lead citrate; then, they were observed via a transmission electron microscope, images were captured, and structures were analyzed.

### 2.9. Immunohistochemistry Staining (IHC)

Fresh tissues fixed in 4% paraformaldehyde (Cat#P0099, Beyotime, China) were dehydrated, cleared, and paraffin-embedded; 4–5 μm sections on polyline slides were baked at 60 °C, then dewaxed, hydrated, and PBS-washed. Then, they were incubated with mucin 5AC (MUC5AC) antibodies (Cat#20725-1-AP, Proteintech, China), rewarmed, PBS-washed, and then HRP-secondary antibodies (Cat#RS0008, Immunoway, USA) were added. They were then PBS-washed and fresh DAB was developed for 3–10 min, followed by hematoxylin counterstaining, 1% HCl-ethanol differentiation, and bluing. Finally, they were dehydrated, cleared, mounted, microscopically observed, and quantified via Image-ImageJ (version 1.53t).

### 2.10. Hematoxylin-Eosin Staining (H&E)

Paraffin sections were dewaxed in xylene, hydrated through gradient ethanol, stained with hematoxylin for 5–10 min, differentiated with 1% hydrochloric acid-ethanol, and blued with tap water. Then they were stained with eosin for 1–3 min, dehydrated with gradient ethanol, cleared in xylene, and mounted with neutral gum. They were observed under a light microscope.

### 2.11. Alcian Blue-Periodic Acid-Schiff Staining (AB-PAS)

Dewaxed and hydrated sections were stained with alcian blue solution (pH 2.5) for 30 min, rinsed, oxidized with periodic acid for 5–10 min, rinsed again, then treated with Schiff reagent for 15–30 min. Then they were counterstained with hematoxylin lightly, dehydrated, cleared, and mounted. Acid mucins appeared blue and neutral mucins magenta.

### 2.12. Masson’s Trichrome Staining (Masson)

Dewaxed and hydrated sections were stained with Weigert’s iron hematoxylin for 5–10 min, rinsed, then stained with Biebrich scarlet-acid fuchsin for 5–10 min. After differentiation with phosphomolybdic acid solution, they were stained with aniline blue for 5–10 min, dehydrated quickly, cleared, and mounted. Collagen fibers appeared blue, cytoplasm red, and nuclei black.

### 2.13. Prussian Blue Staining (Enhance with DAB)

Paraffin sections were dewaxed and then transferred to absolute ethanol I and absolute ethanol II for 5 min each, respectively. The staining solution was prepared by mixing hydrochloric acid and potassium ferrocyanide at a volume ratio of 1:1, and the sections were stained with this solution for 1 h. Subsequently, a DAB stock solution was mixed with the diluent at a volume ratio of 1:50, and the sections were stained with the prepared DAB working solution for 5–10 min. After that, the sections were stained with nuclear fast red for 3–5 min. Finally, the sections were subjected to dehydration and mounting procedures.

## 3. Results

### 3.1. Cold Exposure Aggravated AAI Symptoms

**Figure 1:**
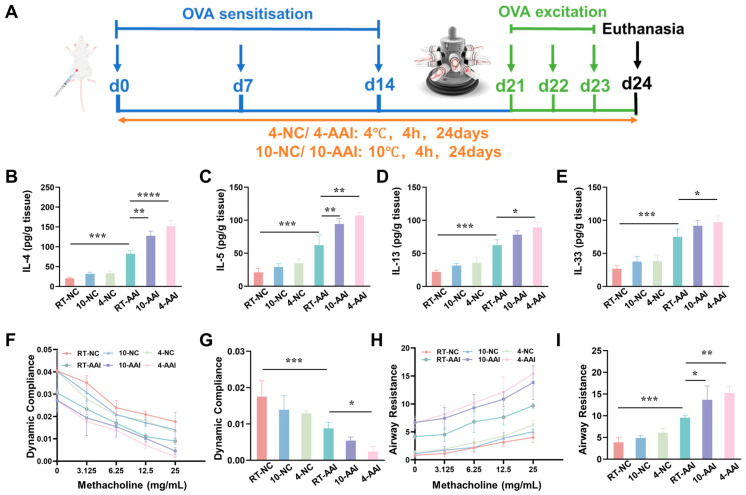
The influence of cold exposure on AAI symptoms. (**A**) Schematic diagram of the establishment of the in vivo model along with the corresponding treatment protocol. The blue arrows point to the time point of OVA sensitization, the green arrows point to the time point of OVA activation, and the black arrow points to the time point of euthanasia sampling. (**B**–**E**) Expressions of IL-4, IL-5, IL-13, and IL-33 in lung after different treatments (*n* = 6). (**F**) The modifications in lung dynamic compliance in response to various treatments. (**G**) The variations in lung dynamic compliance following administration of 25 mg/mL methacholine (*n* = 6). (**H**) The variations in respiratory system resistance in response to various treatments. (**I**) The alterations in respiratory system resistance following treatment with 25 mg/mL methacholine (*n* = 6), * *p* < 0.05; ** *p* < 0.01; *** *p* < 0.001, **** *p* < 0.0001.

To explore whether cold exposure aggravates AAI symptoms, a mouse model was established and operations were performed following the protocol shown in Figure 1A. The inflammatory cytokine levels, cytokine mRNA expression, and lung function were examined. As illustrated in Figure 1B–E, compared with the RT-NC group, all inflammatory cytokines in the RT-AAI group were significantly elevated, confirming the successful establishment of the model. In contrast, although the 10-NC and 4-NC groups showed an upward trend in inflammatory cytokines, no statistically significant differences were observed. When compared with the RT-AAI group, most inflammatory cytokine levels in both the 10-AAI and 4-AAI groups were significantly increased; notably, IL-33 expression in the 10-AAI group exhibited an upward trend relative to the RT-AAI group without reaching statistical significance, which may be attributed to individual variability. Additionally, a similar trend was observed in the relative mRNA expression levels of these cytokines across all groups (Figure S1). Regarding lung function, dynamic compliance (Cdyn) in all groups decreased with increasing methacholine concentration. At a methacholine concentration of 25 mg/mL, mice in the 4-AA group exhibited the lowest Cdyn among all groups, with a statistically significant difference compared to the RT-AAI group (Figure 1F,G). As shown in Figure 1H,I, the respiratory resistance (RI) of mice in all groups gradually increased with escalating methacholine doses. At a methacholine concentration of 25 mg/mL, RI reached its maximum value. Among these groups, both the 10-AAI and 4-AAI groups exhibited statistically significant differences in airway resistance compared to the RT-AAI group, with the 4-AAI group showing a more pronounced difference.

### 3.2. Cold Exposure Aggravated Pathological Lung Damage

**Figure 2:**
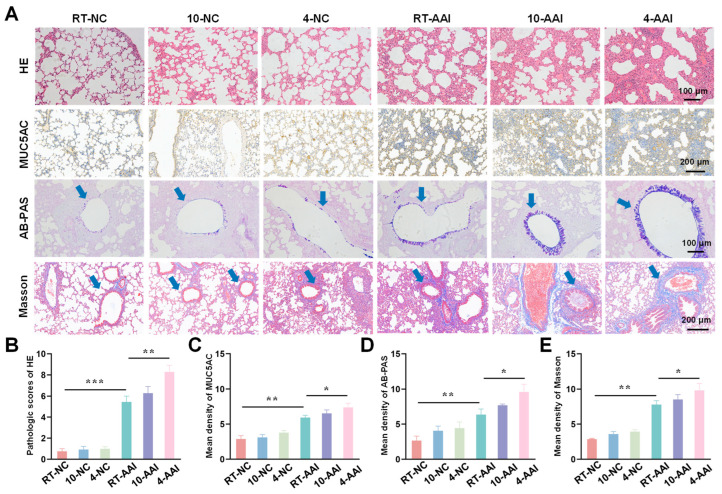
Histopathological alterations and quantitative analysis in lung tissues of mice from different groups. (**A**) Representative images of lung tissue sections stained with H&E, MUC5AC, AB-PAS, and Masson trichrome staining in each group. The blue arrow points to the airway. Scale bars: 100 μm for H&E and AB-PAS; 200 μm for MUC5AC and Masson (*n* = 6). (**B**–**E**) The statistical analysis of (**A**) (*n* = 6). * *p* < 0.05; ** *p* < 0.01; *** *p* < 0.001.

To investigate the effect of cold exposure on pathological changes in lung tissues of AAI mice, a comprehensive evaluation was performed using H&E staining, MUC5AC staining, Alcian AB-PAS staining, and Masson staining. As shown in Figure 2, H&E staining results revealed that there were no significant morphological changes in lung tissue sections of the 10-NC and 4-NC groups compared with the NC group. In contrast, all three AAI mouse groups exhibited prominent pathological alterations, including airway-specific changes (bronchiole wall thickening, goblet cell hyperplasia in airway epithelium) and pan-lung changes (alveolar septa thickening, alveolar fusion). Among all groups, the 4-AAI group showed the most severe pathological changes. Results of MUC5AC staining and AB-PAS staining showed that, compared with the NC group, the 10-NC and 4-NC groups exhibited no significant differences in MUC5AC expression or mucus expression. In contrast, the RT-AAI, 10-AAI, and 4-AAI groups had significantly increased expression of airway mucus. Additionally, similar findings were observed in Masson staining: the 4-AAI group showed the most prominent collagen deposition, and this deposition was significantly higher compared with that in the RT-AAI group. In summary, the aforementioned results collectively indicated that cold exposure could promote the expression of Th2-type cytokines, impair lung function, and exacerbate the symptoms of AAI. Particularly, the effect of 4 °C stimulation on AA was more pronounced compared with that of 10 °C stimulation. It was worth noting that histopathological analysis revealed obvious bronchial wall and peribronchial specific pathological changes. In the RT-AAI group, the abnormalities were mainly concentrated in the bronchi and the areas around the bronchi. Cold exposure further amplified this specificity: Compared with the RT-AA group, the 10-AAI group had intensified collagen deposition around the bronchi and hyperplasia of bronchial epithelial cup cells, while the 4-AAI group exhibited more severe but still airway-centered damage.

### 3.3. Cold Exposure Promoted Cell Death in AAI Mouse Lung Tissue via Ferroptosis

**Figure 3:**
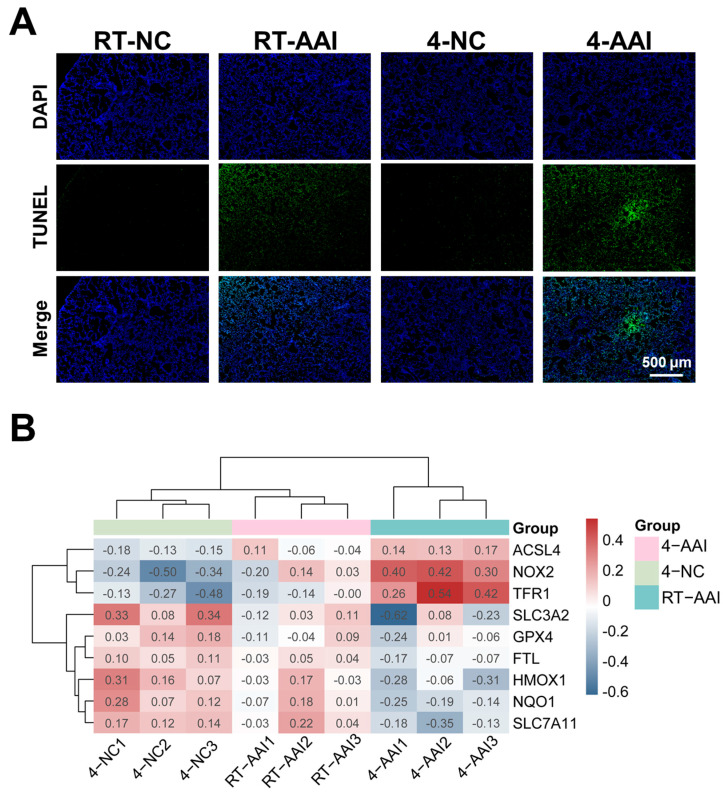
Analysis of cell death patterns and ferroptosis-related gene expression. (**A**) Detection of cell death by TUNEL staining (*n* = 6). (**B**) Clustering heatmap of ferroptosis-related gene expression in mice of each group (*n* = 6). The data used for the heatmap underwent normalization, so the values displayed in the heatmap were normalized rather than actual measured data.

Given that cold exposure is a well-documented environmental trigger for AAI exacerbation, and ferroptosis has been implicated as a critical regulated cell death pathway in various respiratory pathologies. Whether cold exposure modulated lung tissue cell death in a mouse model of AAI was investigated. As shown in Figure 3A and Figure S2, compared with the RT-NC group the cell death rate of lung tissues in the RT-AAI group was significantly increased, with a more pronounced increase in the 4-AAI group. In contrast there was no significant change in the cell death rate of lung tissues in the 4-NC group. These findings indicated that cold exposure exacerbates cell death in the lung tissues of AAI mice. To explore the potential mechanism by which cold exposure promotes cell death, a PCR array was employed to screen for cell death modalities in mouse lung tissues (Figure S3A). The result showed that the expression of multiple death-related genes varied across different groups; however, only the ferroptosis-related genes exhibited a consistent expression trend (Figure S3B and Figure 3B). This suggested that ferroptosis was likely a key underlying mechanism through which cold exposure exacerbates AAI.

### 3.4. Cold Exposure Altered the Expression of Key Ferroptosis-Related Markers

**Figure 4:**
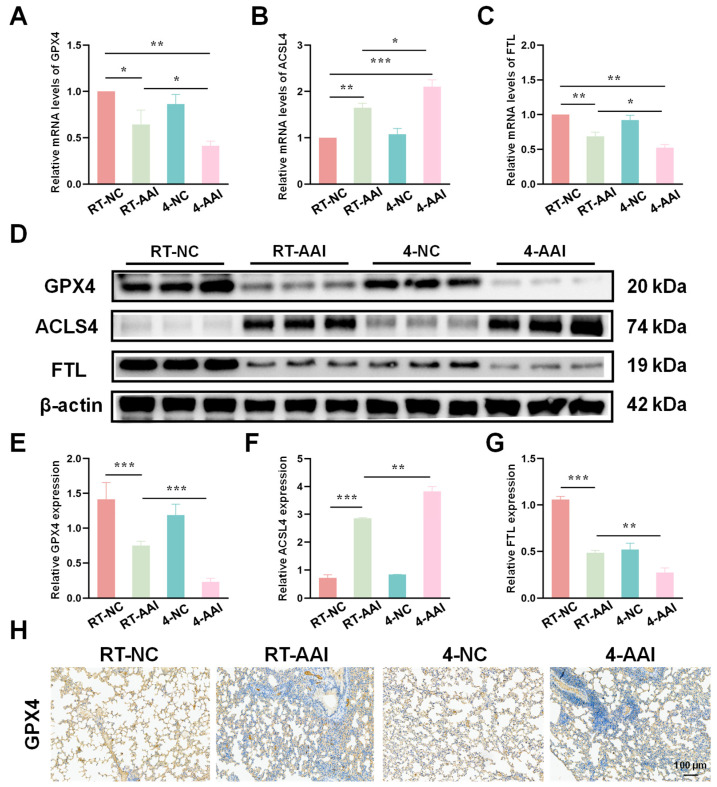
Effects of different treatments on the expression of ferroptosis-related genes and proteins in lung tissue. (**A**–**C**) The relative mRNA expressions of GPX4, ACSL4, and FTL in lung tissue after different treatments (*n* = 6). (**D**) Representative Western blot bands of GPX4, ACSL4, FTL, and β-actin (loading control) in lung tissue (*n* = 6). (**E**–**G**) Quantitative analysis of relative protein expression levels of GPX4, ACSL4, and FTL (*n* = 6). (**H**) IHC staining of GPX4 in lung tissue (*n* = 6). The scale bar indicated 100 μm. * *p* < 0.05; ** *p* < 0.01; *** *p* < 0.001.

Since the PCR array results preliminarily suggested that ferroptosis might serve as the key mechanism underlying cold exposure-exacerbated AAI, further experiments were conducted to verify the hypothesis by detecting the expression of key ferroptosis-related molecules at both transcriptional and translational levels. As shown in Figure 4A–C, qRT-PCR results revealed that compared with the RT-NC group, the mRNA level of GPX4 was decreased in the RT-AAI group and further reduced in the 4-AAI group. And there was no significant change in the 4-NC group. ACSL4 protein expression exhibited the opposite pattern; the mRNA level of ACSL4 was elevated in the RT-AAI group, and more significantly increased in the 4-AAI group. The expression trend of FTL in each group was similar to that of GPX4. These findings were further confirmed by protein expression analyses (Figure 4D–G), where similar trends were observed in Western blot assays. After cold exposure, compared with RT-AAI, the expressions of GPX4 and FTL proteins further decreased in the 4-AA group, while ACSL4 further increased. IHC staining (Figure 4H and Figure S3) further confirmed the changes in GPX4 distribution and expression in lung tissue, with obvious differences among the groups, which was in line with the results of qRT-PCR and Western blot. These findings suggested that cold exposure alters the expression levels of key ferroptosis-related proteins, implying that the exacerbation of AAI under cold conditions might be mechanistically linked to the induction of ferroptosis.

### 3.5. Cold Exposure Induced Characteristic Features of Ferroptosis

**Figure 5:**
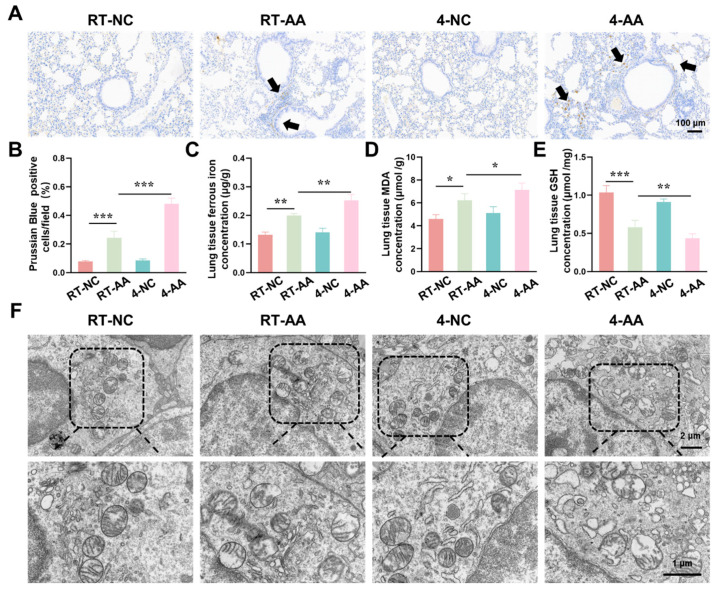
Assessment of ferroptosis-related indicators in lung tissue. (**A**) Prussian Blue staining of lung tissue (*n* = 6). The scale bar indicated 100 μm. Black arrows indicated Prussian Blue-positive cells. (**B**) Quantification of Prussian Blue-positive cells per field (*n* = 6). (**C**) Concentration of ferrous ions in lung tissue (*n* = 6). (**D**) Concentration of MDA in lung tissue (*n* = 6). (**E**) Concentration of GSH in lung tissue (*n* = 6). (**F**) TEM images of lung tissue ultrastructure (*n* = 6). The scale bars respectively indicated 2 μm and 1 μm, * *p* < 0.05; ** *p* < 0.01; *** *p* < 0.001.

Having confirmed that cold exposure modulated the expression of key ferroptosis-related genes and proteins in AAI mice, we next aimed to comprehensively evaluate the actual ferroptosis status in lung tissues by detecting its canonical hallmarks, including iron accumulation, lipid peroxidation, antioxidant capacity changes, and characteristic ultrastructural alterations. As shown in Figure 5A,B, the percentage of Prussian Blue-positive cells (indicating iron deposition) was significantly higher in the RT-AAI group than in the RT-NC group, and the 4-AAI group had a remarkably greater proportion of positive cells than the 4-NC group, suggesting enhanced iron accumulation. When comparing RT-AAI and 4-AAI, the 4-AAI group exhibited a significantly higher percentage of Prussian Blue-positive cells than the RT-AAI group, indicating more pronounced iron deposition in 4-AAI. Consistent with this, the concentration of ferrous iron in lung tissue (Figure 5C) was elevated in the RT-AAI group compared to the RT-NC group and in the 4-AAI group relative to the 4-NC group; moreover, the 4-AA group showed a significantly higher ferrous iron concentration than the RT-AAI group. MDA, a key marker of lipid peroxidation, was measured to evaluate oxidative stress, and as shown in Figure 5D, the lung tissue MDA concentration in the RT-AAI group was significantly higher than that in the RT-NC group, with the 4-AAI group also having a notably higher MDA level compared to the 4-NC group; the 4-AAI group also exhibited a significantly higher MDA concentration than the RT-AAI group, indicating that cold exposure exacerbated the degree of lipid peroxidation in AAI mice. GSH as a critical intracellular antioxidant was also assessed. The GSH concentration in lung tissue (Figure 5E) was significantly lower in the RT-AAI group than in the RT-NC group and in the 4-AAI group compared to the 4-NC group. Additionally, the 4-AA group had a significantly lower GSH concentration than the RT-AAI group, reflecting that cold exposure impaired the antioxidant defense function in AAI mice. TEM was utilized to observe ultrastructural alterations in lung tissue (Figure 5F). In the RT-NC and 4-NC groups, the cellular ultrastructure of lung tissue appeared relatively normal, with organelles maintaining structural integrity. In contrast, distinct ferroptosis-related morphological alterations were observed in the RT-AAI and 4-AAI groups. These characteristic changes included rupture of mitochondrial membranes, reduced or absent mitochondrial cristae density, and mitochondrial swelling—all of which are well-recognized ultrastructural hallmarks of ferroptosis. Notably, the extent of these ultrastructural abnormalities was more severe in the 4-AAI group than in the RT-AAI group. This observation further supported that cold exposure enhanced the intensity of ferroptosis in AAI mice.

To disentangle the respective contributions of allergic sensitization, cold exposure, and their interaction across endpoints, we summarized the main effects and interaction terms using a two-way factorial analysis. As shown in Table 1, allergic sensitization exerted a significant main effect on most inflammatory, functional, and histopathological parameters, while cold exposure showed an additional and often stronger main effect across multiple endpoints. Importantly, significant sensitization × cold interaction effects were observed for several key outcomes, indicating that cold exposure disproportionately exacerbates disease severity under allergic conditions.

### 3.6. Fer-1 Ameliorated Cold Exposure-Induced Exacerbation of Inflammation and Lung Function

**Figure 6:**
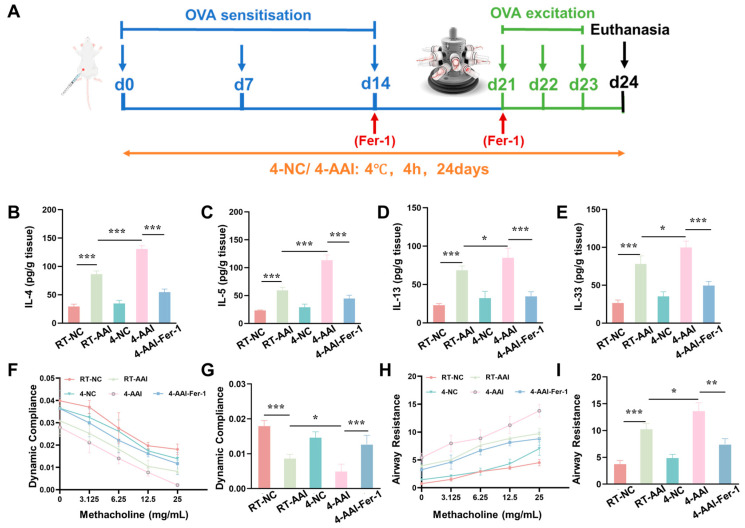
The increase of AAI inflammatory factors and the deterioration of lung function caused by cold exposure were alleviated by Fer-1 treatment. (**A**) Schematic diagram of the establishment of the in vivo model along with the corresponding treatment protocol. (**B**–**E**) Expressions of IL-4, IL-5, IL-13, and IL-33 in lung after different treatments (*n* = 6). (**F**) The modifications in lung dynamic compliance in response to various treatments. (**G**) The variations in lung dynamic compliance following administration of 25 mg/mL methacholine (*n* = 6). (**H**) The variations in respiratory system resistance in response to various treatments. (**I**) The alterations in respiratory system resistance following treatment with 25 mg/mL methacholine (*n* = 6), * *p* < 0.05; ** *p* < 0.01; *** *p* < 0.001.

Given that our previous findings collectively confirmed that cold exposure exacerbates AAI in mice by promoting ferroptosis in lung tissues, we next sought to validate the causal link between ferroptosis and cold-induced AAI aggravation through an intervention approach. Specifically, we employed Fer-1, a well-characterized specific inhibitor of ferroptosis, to explore whether suppressing ferroptosis could alleviate the exacerbation of AAI symptoms triggered by cold exposure. A mouse model was established and operations were performed following the protocol shown in Figure 6A. Compared with the RT-NC group, the concentrations of Th2 cytokines (IL-4, IL-5, IL-13) and IL-33 in lung tissue were elevated in the RT-AAI group, and further increased in the 4-AA group; administration of Fer-1 reversed these increases (Figure 6B–E). qRT-PCR results (Figure S4) exhibited consistent trends at the mRNA level. Compared with the RT-NC group, relative mRNA levels of IL-4, IL-5, IL-13, and IL-33 were up-regulated in the RT-AAI group, and more prominently up-regulated in the 4-AA group, while Fer-1 treatment attenuated these up-regulations. Assessment of lung function (Figure 6F–I) revealed that dynamic lung compliance decreased and airway resistance increased in a methacholine-dose-dependent manner in the RT-AAI group compared to the RT-NC group, whereas the 4-AAI group displayed worsened compliance and heightened resistance. Notably, Fer-1 administration in the 4-AAI-Fer-1 group mitigated the impairment of dynamic compliance and the elevation of airway resistance. In addition, there were no significant changes in the overall cytokine levels and lung function parameters of mice in the 4-NC group compared with those in the RT-NC group. In summary, these data indicated that in this mouse model, Fer-1 treatment could improve, to a certain extent, the aggravation of symptoms in AAI mice caused by cold exposure.

### 3.7. Fer-1 Attenuated Cold Exposure-Induced Histopathological Damage

**Figure 7:**
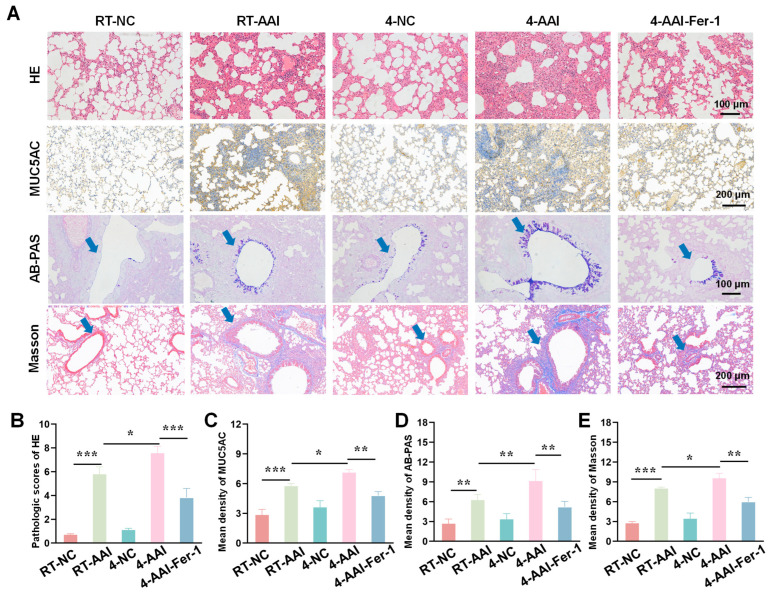
AAI lung pathological tissue damage caused by cold exposure was alleviated by Fer-1 treatment. (**A**) Representative images of lung tissue sections stained with HE, MUC5AC, AB-PAS, and Masson trichrome staining in each group (*n* = 6). The blue arrow points to the airway. Scale bars: 100 μm for HE and AB-PAS; 200 μm for MUC5AC and Masson. (**B**–**E**) The statistical analysis of (**A**) (*n* = 6). * *p* < 0.05; ** *p* < 0.01; *** *p* < 0.001.

Building on the evidence that Fer-1 mitigated cold exposure-induced exacerbation of AAI-related cytokine dysregulation and lung function impairment, we further sought to assess the therapeutic effect of ferroptosis inhibition at the tissue structural level. To this end, we employed a series of histopathological staining techniques to systematically evaluate the extent of lung tissue damage, inflammatory infiltration, mucus hypersecretion, and collagen deposition-key pathological features of AAI-across different experimental groups. As shown in Figure 7A, HE staining revealed that compared with the RT-NC group, lung tissues in the RT-AAI group exhibited more severe inflammatory cell infiltration and structural disruption; the 4-AAI group showed further aggravated pathological changes, while no significant changes were observed in the 4-NC group, and the 4-AAI-Fer-1 group displayed alleviated pathology. MUC5AC, AB-PAS staining, and Masson staining were used to assess mucus production and collagen deposition, respectively. Quantitative analysis (Figure 7B–E) demonstrated that the RT-AAI group had significantly higher HE pathological scores, mean density of MUC5AC, mean density of AB-PAS, and mean density of Masson compared with the RT-NC group. The 4-AAI group showed even higher values in these parameters, whereas the 4-NC group had no significant changes, and Fer-1 treatment in the 4-AAI-Fer-1 group notably reduced these elevated indices. Collectively, these results indicated that cold exposure exacerbated AAI-related histopathological damage in mice, and Fer-1 could mitigate such damage.

### 3.8. Fer-1 Mitigated Cold Exposure-Induced Ferroptosis-Related Protein Dysregulation

**Figure 8:**
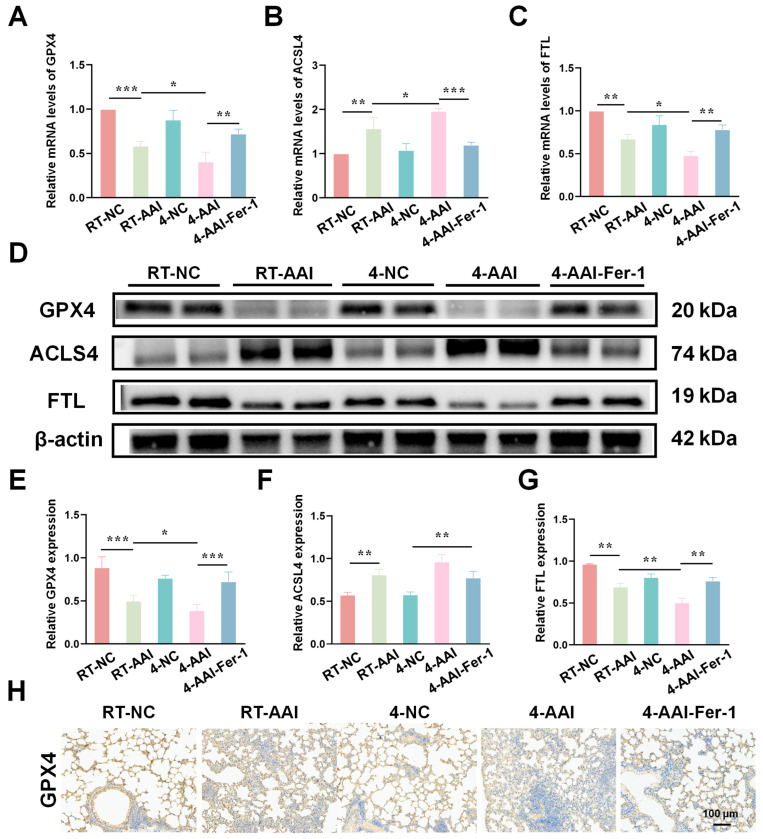
The expression of genes related to ferroptosis in the lung tissues of AAI mice caused by cold exposure was affected by Fer-1 treatment. (**A**–**C**): Relative mRNA levels of GPX4 (**A**), ACSL4 (**B**), and FTL (**C**) in lung tissues of various groups (*n* = 6). (**D**) Representative Western blot bands of GPX4, ACSL4, FTL, and β-actin (loading control) in lung tissues (*n* = 6). (**E**–**G**) Quantitative analysis of relative protein expression levels of GPX4 (**E**), ACSL4 (**F**), and FTL (**G**) normalized to β-actin (*n* = 6). (**H**) IHC staining of GPX4 in lung tissue (*n* = 6). The scale bar indicated 100 μm. * *p* < 0.05; ** *p* < 0.01; *** *p* < 0.001.

To elucidate the regulatory effects of different interventions on ferroptosis-related molecules in lung tissues, the mRNA and protein expression of key ferroptosis mediators were assessed. As shown in Figure 8A–C, qRT-PCR results indicated that compared with the RT-NC group, the mRNA level of GPX4 was decreased in the RT-AAI group, and further reduced in the 4-AAI group; Fer-1 treatment in the 4-AAI-Fer-1 group reversed this reduction. In contrast, the mRNA level of ACSL4 was elevated in the RT-AAI group, and more significantly increased in the 4-AAI group, with Fer-1 attenuating this elevation. The mRNA level of FTL showed a decrease in the RT-AA group, a notable decrease in the 4-AAI group, and a recovery upon Fer-1 treatment. Western blot analysis (Figure 8D–G) exhibited consistent trends at the protein level: GPX4 protein expression was down-regulated in the RT-AAI group, down-regulated again in the 4-AAI group, and up-regulated in the 4-AAI-Fer-1 group. ACSL4 protein expression displayed the opposite pattern, being up-regulated in the RT-AAI group, further up-regulated in the 4-AAI group, and down-regulated in the 4-AAI-Fer-1 group. FTL protein expression was reduced in the RT-AAI group, markedly decreased in the 4-AAI group, and increased in the 4-AAI-Fer-1 group. IHC staining (Figure 6H and Figure S4) further confirmed the changes in GPX4 distribution and expression in lung tissues, with distinct differences among the groups, which was consistent with the results of qRT-PCR and Western blot. Collectively, these findings suggested that different treatment regimens exert distinct regulatory effects on ferroptosis-related gene and protein expression in lung tissues, and Fer-1 could modulate these effects.

### 3.9. Fer-1 Alleviated Cold Exposure-Induced Ferroptosis-Related Indicators Dysregulation

**Figure 9:**
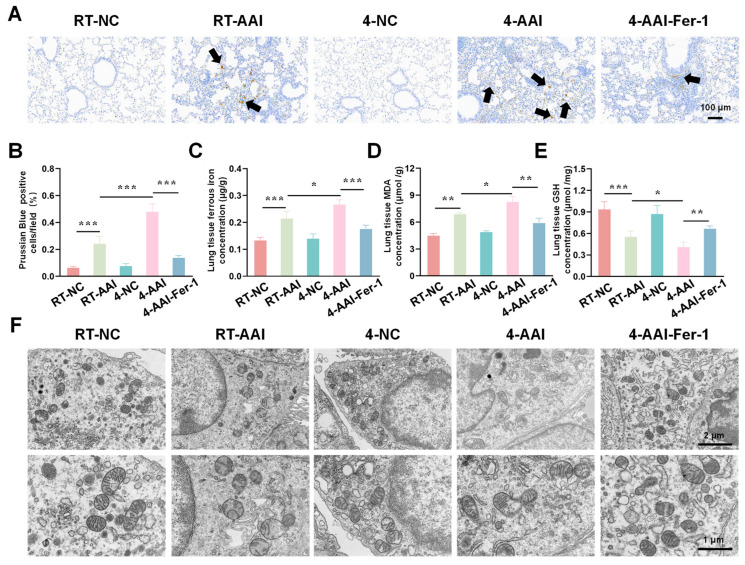
The ferroptosis-related indicators of cold exposure aggravating AAI were improved by Fer-1 treatment. (**A**) Representative Prussian Blue staining images of lung tissues from each group (black arrows point to Prussian Blue-positive cells) (*n* = 6). The scale bar indicated 100 μm. (**B**) Quantitative analysis of Prussian Blue-positive cells. (**C**–**E**) Measurements of lung tissue ferrous iron concentration (**C**), MDA level (**D**), and GSH content (**E**) (*n* = 6). (**F**) TEM images of lung tissues in each group (*n* = 6). The scale bars respectively indicated 2 μm and 1 μm. * *p* < 0.05; ** *p* < 0.01; *** *p* < 0.001.

To investigate iron metabolism, oxidative stress, and ultrastructural alterations in lung tissues after Fer-1 treatment, a series of analyses were conducted. As shown in Figure 9A, B, Prussian Blue staining revealed that compared with the RT-NC group, the percentage of Prussian Blue-positive cells was increased in the RT-AAI group, and further elevated in the 4-AAI group. Fer-1 treatment in the 4-AAI-Fer-1 group reduced this iron deposition. Regarding oxidative stress indicators, the concentration of ferrous iron (Figure 9C) and MDA (Figure 9D) was higher in the RT-AAI group than in the RT-NC group, further increased in the 4-AAI group, and decreased in the 4-AAI-Fer-1 group. The 4-NC group did not show significant changes. In contrast, compared with the RT-NC group, the GSH content (Figure 9E) was lower in the RT-AAI group, further reduced in the 4-AA group, and increased in the 4-AAI-Fer-1 group. TEM images (Figure 9F) showed that the mitochondrial structure in the lung tissue of the RT-NC group was normal. The RT-AAI group exhibited signs of ferroptosis, such as the reduction of mitochondrial cristae. The 4-AAI group displayed more severe ultrastructural disruption, and the 4-AAI-Fer-1 group showed alleviated damage. Collectively, these results suggested that cold exposure exacerbated iron accumulation and oxidative stress in lung tissues of AAI mice, and Fer-1 could mitigate these adverse changes.

## 4. Discussion

AAI is a prevalent chronic respiratory condition significantly influenced by environmental factors, with cold exposure being a recognized trigger for exacerbations. Numerous epidemiological studies have demonstrated a clear association between extreme temperatures. Among them, cold exposure has long been regarded as a key factor leading to acute exacerbation of the disease [17,18]. Cold exposure-induced AAI exacerbations are typically linked to heightened airway inflammation, mucus hypersecretion, and impaired lung function [19]. However, the molecular mechanism between cold exposure and AAI exacerbation has not been fully elucidated, which limits the development of targeted interventions for such climate-related disease burdens.

Cold exposure can trigger a series of physiological and pathological changes in the respiratory system, and these changes work together to exacerbate AAI. Firstly, cold air directly stimulates the smooth muscles of the airways, causing vasoconstriction and increased muscle tone-this effect is particularly significant in AAI patients who already have airway hyperresponsiveness [20]. This will lead to an immediate increase in airway resistance, as we observed in the mouse model, a decrease in Cdyn and an increase in RI. Secondly, cold exposure undermines the integrity of the airway epithelial barrier: Low temperatures can damage the tight junction function of epithelial cells, reduce the efficiency of mucosal ciliary clearance, and promote the death of epithelial cells, thereby creating a “leaking” airway microenvironment that provides favorable conditions for allergen penetration and microbial colonization [17]. The histopathological analysis of this study revealed that cold-exposed AAI mice had severe epithelial damage, goblet cell proliferation and hypersecretion of mucus (confirmed by increased MUC5AC expression and AB-PAS staining results). Thirdly, cold exposure amplifies type 2 inflammatory responses: Our data show that cold stress (especially at 4 °C) significantly upregulates the expression of helper Th2-related cytokines (IL-4, IL-5, IL-13) and the alarm hormone IL-33 in lung tissue. Among them, IL-33 plays a key role in activating type 2 innate lymphocytes (ILC2s) and Th2 cells, forming a positive feedback loop that maintains airway inflammation [21].

Ferroptosis, an iron-dependent form of regulated cell death characterized by lipid peroxidation, iron accumulation, and mitochondrial damage, has emerged as a critical player in the pathophysiology of various pulmonary diseases, including acute lung injury and COPD [22,23,24]. Increasing evidence suggests that ferroptosis may contribute to airway epithelial damage in AAI, exacerbating inflammation and impairing lung function. Recent studies have highlighted the involvement of altered iron metabolism and elevated lipid peroxidation products in AAI, further implicating ferroptosis in the disease’s progression [25,26]. In our study, we observed that cold exposure in AAI mice triggered the hallmark features of ferroptosis in lung tissues, including elevated ACSL4 expression, reduced GPX4 and FTL levels, increased iron deposition, and enhanced lipid peroxidation, as indicated by increased MDA levels. Importantly, inhibition of ferroptosis using Fer-1 significantly alleviated the exacerbation of AAI symptoms, including reductions in airway inflammation, mucus hypersecretion, airway resistance, and histopathological damage. These results confirmed that ferroptosis was a key mediator linking cold exposure to AAI aggravation, highlighting its potential as a therapeutic target. However, it was observed that the efficacy of Fer-1 was not uniform in all pathological manifestations of AAI aggravated by cold exposure. While Fer-1 administration robustly reversed the core biochemical signatures of ferroptosis, its impact on other endpoints was more moderate. Notably, the improvements in lung function parameters, though statistically significant, were partial. This spectrum of efficacy suggests that ferroptosis acts as a predominant driver of acute epithelial injury, oxidative stress, and the secretory phenotype in the cold-stressed airway, processes directly linked to mucus pathology. In contrast, the pathways leading to sustained airway dysfunction and fibrotic remodeling appear to be more complex and multifactorial, involving mechanisms beyond acute ferroptosis that are not fully corrected by its short-term inhibition. Consequently, the modest restoration of lung function aligns with Fer-1’s role as a specific inhibitor targeting a key upstream pathogenic event rather than a broad-spectrum agent. This understanding positions the inhibition of ferroptosis not as a standalone remedy for immediate functional recovery, but as a promising adjunctive strategy. It holds particular potential for mitigating the epithelial damage and mucus hypersecretion that characterize disease exacerbations, thereby complementing existing therapies within a combined management approach for environmental trigger-induced airway inflammation. While our study confirmed ferroptosis as the dominant cell death pathway linking cold exposure to AAI exacerbation, we acknowledge that apoptosis and necrosis may also be involved in the pathological process—consistent with the preliminary observations from PCR array and TUNEL staining. However, these modalities were not fully characterized in the current work, as our focus was on validating the ferroptosis-mediated mechanism. Future studies will build on the current model to explore the crosstalk between ferroptosis, apoptosis, and necrosis which may provide a more comprehensive understanding of the molecular network underlying cold-induced AAI aggravation and inform the development of multi-target therapeutic strategies.

Several limitations of this study should be acknowledged. First, our investigations were conducted in an OVA-induced mouse model of AAI. While this model recapitulated several key features of human AAI—such as Th2 inflammation, airway hyperresponsiveness, and remodeling—it represented a pan-lung inflammatory response rather than a disease confined strictly to the airways. A second limitation was the absence of OVA-specific bronchoprovocation testing, which would have allowed us to directly assess antigen-driven airway responsiveness—a defining feature of AAI. Our use of methacholine-induced non-specific AHR captured cumulative airway dysfunction but did not distinguish immune-mediated responses to OVA. Future studies should incorporate OVA-specific bronchoprovocation to validate whether cold exposure exacerbates antigen-specific AHR and whether ferroptosis inhibition modulates this pathway, particularly in humanized models or in vitro systems using primary human airway cells. Third, although we focused on ferroptosis, cold exposure may concurrently engage other forms of regulated cell death (e.g., apoptosis, necroptosis), and potential crosstalk among these pathways warrants further exploration. Further research is needed to explore whether ferroptosis interacts with other forms of regulated cell death. While challenges remain in translating these findings into clinical practice, our study establishes a “cold exposure-ferroptosis-AAI exacerbation” axis, uncovering ferroptosis as a link between environmental cold exposure and allergic airway pathology. The reversal of cold exposure-induced airway inflammation and dysfunction by Fer-1 not only validates ferroptosis as a promising therapeutic target but also opens new avenues for developing interventions aimed at mitigating the climate-related burden of AAI and similar allergic respiratory conditions.

## 5. Conclusions

In summary, our study identified the activation of ferroptosis as a critical pathogenic mechanism underlying cold exposure-induced exacerbation of AAI. The consistent alleviation of inflammation, histological damage, and lung dysfunction upon treatment with Fer-1 solidified ferroptosis as a promising therapeutic target. These findings connected environmental epidemiology with molecular pathophysiology, and indicated that interventions targeting ferroptosis could lead to new strategies for managing AAI exacerbated by climatic factors, laying a new foundation for the personalized treatment of AAI patients.

## Figures and Tables

**Table 1:** Main and interaction effects of allergic sensitization and cold exposure.

Outcome Measure	Sensitization (*p*-Value)	Cold (4 °C) (*p*-Value)	Interaction
IL-4 (Lung)	<0.0001	<0.0001	<0.0001
IL-5 (Lung)	<0.0001	<0.0001	<0.0001
IL-13 (Lung)	<0.0001	<0.0001	0.0353
IL-33 (Lung)	<0.0001	0.0005	0.0525
Cdyn	<0.0001	<0.0001	0.7538
RI	<0.0001	<0.0001	0.0003
Mucus Score	<0.0001	<0.0001	0.0026
HE	<0.0001	<0.0001	<0.0001
GPX4	0.0025	<0.0001	0.1267
Fe2+	0.0611	<0.0001	0.0898
MDA	0.0131	<0.0001	0.0846

*p*-values were derived from two-way ANOVA with allergic sensitization (NC vs. AAI), temperature (RT vs. 4 °C), and their interaction as fixed effects. For each endpoint, the *p*-values for the main effect of sensitization, the main effect of cold exposure, and the sensitization × cold interaction were reported.

## Data Availability

Data will be available on request from the corresponding author.

## Supplementary Materials

The following supporting information can be downloaded at: https://www.mdpi.com/article/10.3390/antiox15010032/s1, Table S1. Sequences of primers used in this study; Figure S1. The relative mRNA expressions of IL-4, IL-5, IL-13 and IL-33 in lung after different treatments (*n* = 6); Figure S2. Quantitative analysis of TUNEL-positive cells in lung tissues among different groups. ** *p*  <  0.01; *** *p*  <  0.001; Figure S3. (A) Heatmap of death mode screening by PCR array (*n* = 6). (B) Expression trends of different death mode-related genes in mice of each group (*n* = 6); Figure S4. Quantitative analysis of GPX4-positive cells in lung tissues among different groups. ** *p * <  0.01; *** *p*  <  0.001; Figure S5. The relative mRNA expressions of IL-4, IL-5, IL-13 and IL-33 in lung after different treatments (*n* = 6); Figure S6. Quantitative analysis of GPX4-positive cells in lung tissues among different groups. ** *p*  <  0.01; *** *p*  <  0.001.
