# Supplementary material for: Cold Exposure Exacerbates Allergic Airway Inflammation via Ferroptosis: Evidence from a Murine Model
Source: Antioxidants (Basel). 2025 Dec 24;15(1):32. doi: 10.3390/antiox15010032 (PMC12838126; doi:10.3390/antiox15010032)
Supplement: Supplementary file 1 [file antioxidants-15-00032-s001.zip › antioxidants-4037665-supplementary.pdf]

Supplementary Materials

Cold exposure exacerbates allergic airway inflammation via ferroptosis: evidence from a murine model

Xiaoping Guo[1,2,6], Chao Wang[1,2], Xin Yu[1,2], Shanshan Zhang[1,4], Haoyu Zheng[1,2], Tianqi Liu[1,5], Zhili Chen[1,5], Guoqiang Wang[1,2,3], Fang Wang*, [1,2,3,4,5]

[1] Department of Pathogen Biology, College of Basic Medical Sciences, Jilin University, Changchun 130021, China.

[2] The Medical Basic Research Innovation Center of Airway Disease in North China, Changchun 130021, China.

[3] Jilin Provincial Key Laboratory of Precision Infectious Diseases, Changchun 130021, China.

[4] Gene Function and Human Disease Model Organism Sharing Service Platform, Changchun 130021, China.

[5] Jilin Provincial International Cooperation Key Laboratory of Pathogen Biology, Changchun 130021, China.

[6] Cross-disciplinary Innovation Center, Jilin University, Changchun, 130021, China.

*Corresponding authors: wf@jlu.edu.cn (F.W.)

Table S1. Sequences of primers used in this study.

Name	Accession Number	Sequences
GAPDH	NM_001289726.2	F: GGTTGTCTCCTGCGACTTCA<br>R: TGGTCCAGGGTTTCTTACTCC
IL-4	NM_021283.2	F: CGGCACAGAGCTATTGATGGG<br>R: CGCATCCGTGGATATGGCTC
IL-5	NM_010558.1	F: TCCTCAACTCCCTGCTACTCTCC<br>R: TGGCTGGCTCTCATTCACACTG
IL-13	NM_008355.3	F: TCTTGCTTGCCTTGGTGGTCTC<br>R: GGGAATCCAGGGCTACACAGAAC
IL-33	NM_001164724.2	F: TCAGGCGACGGTGTGGATGG<br>R: GGAGTAGTCCTTGTCGTTGGCATG

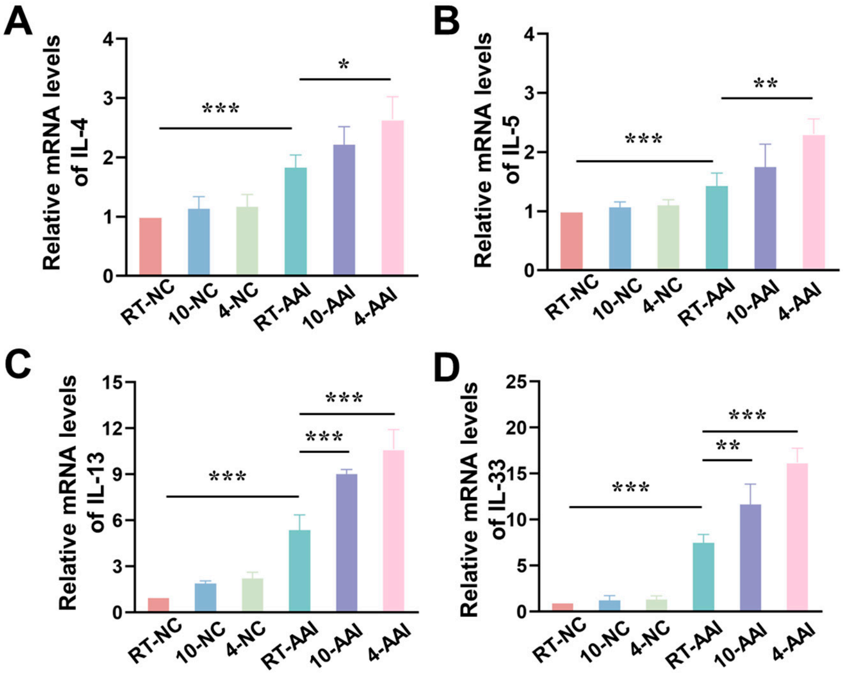

Figure S1. The relative mRNA expressions of IL-4, IL-5, IL-13 and IL-33 in lung after different treatments (*n*=6).

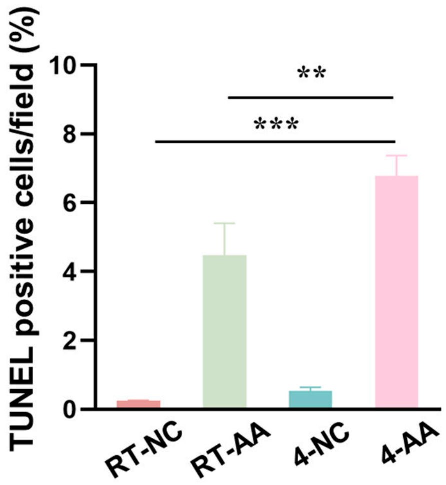

Figure S2. Quantitative analysis of TUNEL-positive cells in lung tissues among different groups. $**p < 0.01$; $***p < 0.001$.

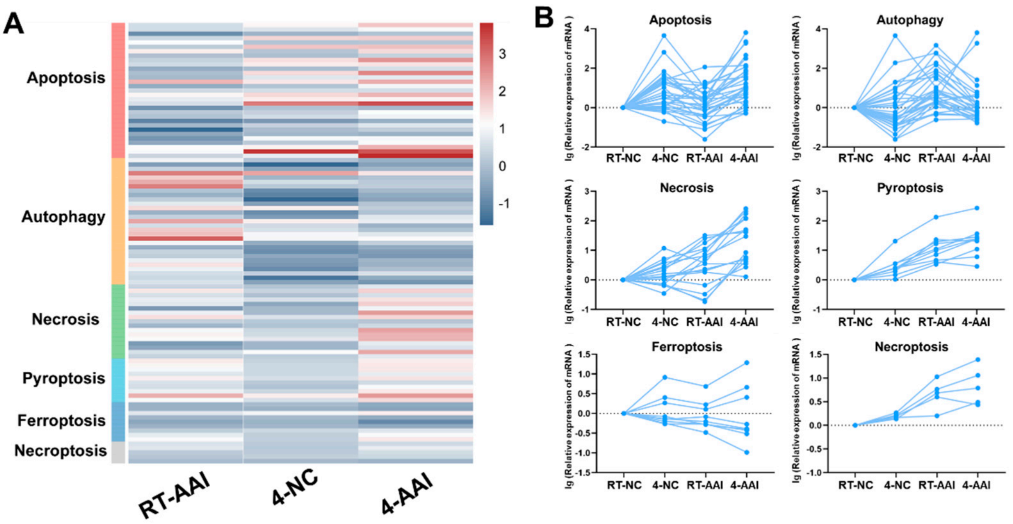

Figure S3. (A) Heatmap of death mode screening by PCR array (*n*=6). (B) Expression trends of different death mode-related genes in mice of each group (*n*=6).

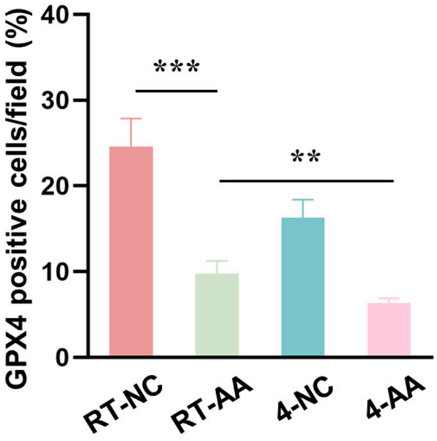

Figure S4. Quantitative analysis of GPX4-positive cells in lung tissues among different groups. **$p < 0.01$; ***$p < 0.001$.

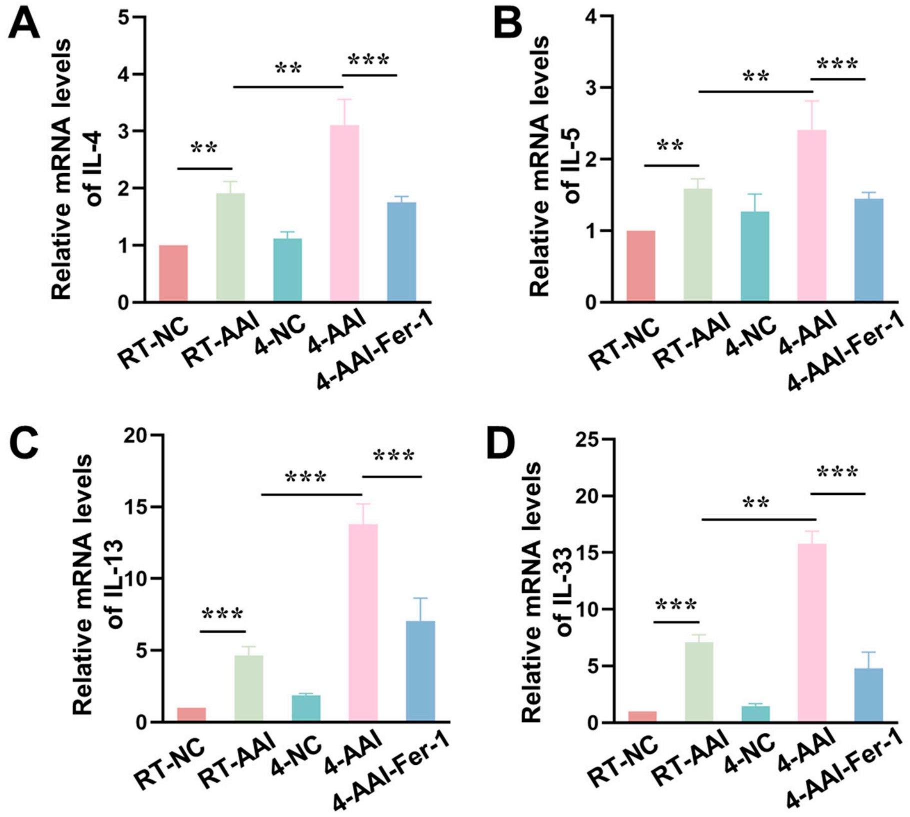

Figure S5. The relative mRNA expressions of IL-4, IL-5, IL-13 and IL-33 in lung after different treatments (*n*=6).

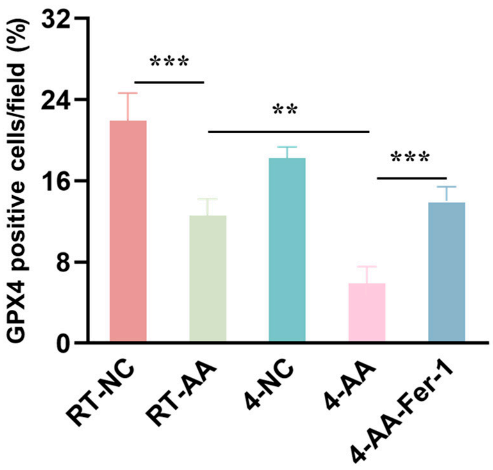

Figure S6. Quantitative analysis of GPX4-positive cells in lung tissues among different groups. ***p* < 0.01; ****p* < 0.001.
